# Dietary supplementation with N-acetylcysteine confers a protective effect on muscle and liver in lipopolysaccharide-challenged piglets

**DOI:** 10.3389/fnut.2024.1458912

**Published:** 2024-09-16

**Authors:** Peng Li, Hancong Zhou, Yuzhu Yang, Mengjun Wu, Di Zhao, Lei Wang, Dan Yi, Yongqing Hou

**Affiliations:** Engineering Research Center of Feed Protein Resources on Agricultural By-Products, Ministry of Education, Hubei Key Laboratory of Animal Nutrition and Feed Science, Wuhan Polytechnic University, Wuhan, Hubei, China

**Keywords:** N-acetylcysteine, weaned piglets, muscle, liver, lipopolysaccharide

## Abstract

*N*-acetylcysteine (NAC) is a well-established antioxidant that offers exciting opportunities for intestinal health in weaned piglets, while the effects of NAC on muscle and liver has not been fully characterized. Therefore, the present study was performed to investigate the effects of dietary supplementation with NAC on muscle and liver in weaned piglets challenged with lipopolysaccharide (LPS). Twenty-four piglets (24-day-old) were randomly assigned to three treatment groups, the piglets in the control (CTR) and LPS- challenged (LPS) groups were fed the basal diet and those in the LPS+ NAC group was fed the basal diet supplemented with 500 mg/kg NAC. The animal trial lasted for 21 days. At the end of the trial, piglets in the LPS and LPS+ NAC groups were injected intraperitoneally with LPS (100 μg/kg body weight) and piglets in the CTR group were administrated with an equal volume of normal saline. 3 h later, the blood was collected and tissue samples were obtained after 6 h of LPS or normal saline treatment. The results showed that the level of IL-1β, and the mRNA levels of C-X-C motif chemokine receptor 3 (*CXCR3*) and interferon-γ (*IFN-*γ) in the liver were up-regulated, and the mRNA levels of insulin-like growth factor 1 (*IGF-1*), total glutathione (T-GSH), and the ratio of total protein to DNA in the liver were decreased under LPS challenge (*P* < 0.05). At the same time, LPS increased the level of H_2_O_2_ and decreased the content of T-GSH and DNA in the longissimus dorsi and gastrocnemius muscles (*P* < 0.05). In addition, the percentage of monocytes and the level of epidermal growth factor (EGF) were down-regulated in the LPS treatment (*P* < 0.05). Interestingly, dietary NAC supplementation reversed the above changes induced by LPS (*P* < 0.05). Furthermore, NAC might alleviate the muscle and liver injury in LPS-challenged piglets by regulating the expression of genes related to the type I interferon signaling pathway, as well as hypoxia inducible factor 1 (*HIF1*) and nuclear factor erythroid-2 related factor 2 (*Nrf-2*). Our findings suggested that dietary supplementation with NAC could benefit the health of muscle and liver in LPS-challenged weaned piglets.

## 1 Introduction

Newly weaned piglets were sensitive to a variety of stressors, which provided an opportunity for the development of weaning syndrome. The immunity of piglets under stress were weak and therefore insufficient to defend against infection by pathogenic microorganisms (1, 2). In addition, the prohibition of antibiotics in feeds also offered an opportunity for the proliferation of pathogenic microorganisms in the gastrointestinal tract of piglets (3). Some pathogenic microorganisms colonized the intestine and secreted perforins or endotoxins to impair the intestinal barrier, and the injured intestine opened the door for bacterial translocation (4). Pathogens and viruses were translocated to various tissues through the bloodstream, causing tissue injury and inflammation (4). Lipopolysaccharide (LPS), a component of the cell wall of Gram-negative bacteria, promoted the secretion of pro-inflammatory cytokines, such as IL-1β, IL-6, and TNF-α (36). In addition, LPS increased the levels of superoxide anion and oxygen free radicals, these pro-inflammatory factors and oxygen free radicals were detrimental to the intestine, liver and muscle (5, 6). Inflammation and oxidative stress not only cause the deterioration of meat quality, but also result in the increase of mortality. This will ultimately negatively affect the production performance of piglets and the economic benefits of breeding enterprises. At present, the nutrient intervention is considered to be an effective measure to alleviate weaning stress and improve production performance and health of piglets.

N-acetylcysteine (**NAC**) is a precursor of L-cysteine and glutathione and was readily absorbed by intestinal epithelial cells (13). The absorbed NAC was transported with the blood to the liver and converted to L-cysteine. Then, L-cysteine was further converted to glutathione (8). Additionally, the ability of NAC to neutralize oxygen radicals and superoxide anions was facilitated by the sulfhydryl group (-SH) in its molecular structure (9). Studies demonstrated that dietary supplementation with NAC contributed to intestinal absorption, barrier and antioxidant function in piglets (10) and that NAC also attenuated LPS-induced inflammatory responses in piglets (11). Our previous study suggested that the growth performance was not affected by dietary supplementary with NAC, interestingly, NAC alleviated LPS-induced intestinal injury in piglets by regulating multiple signaling pathways involving EGFR, PI3K-Akt-mTOR, TLR4-NF-κB, and type I interferon signaling pathways (12). It is generally established that intestine is the main place for the digestion and absorption of nutrients, and it is also the largest immune organ of the body. We hypothesized that NAC helped the intestine, which would further improve the health of other organs, for instance, liver and muscles of the piglets. And our previous study has provided guidance in the general direction (11, 12). However, the effects of NAC on liver and muscle of piglet have not been fully characterized.

We believed it would be interesting to study the effects of NAC on the liver and muscles. This work might provide more comprehensive information on the application of NAC in improving health of piglet and its meat quality in the future. Hence, in the present study, an LPS-stimulated model was used to challenge the liver and muscles of piglets, and the effects of NAC on the muscle and liver of piglets were investigated.

## 2 Materials and methods

### 2.1 Animals and experimental design

**Figure 1:**
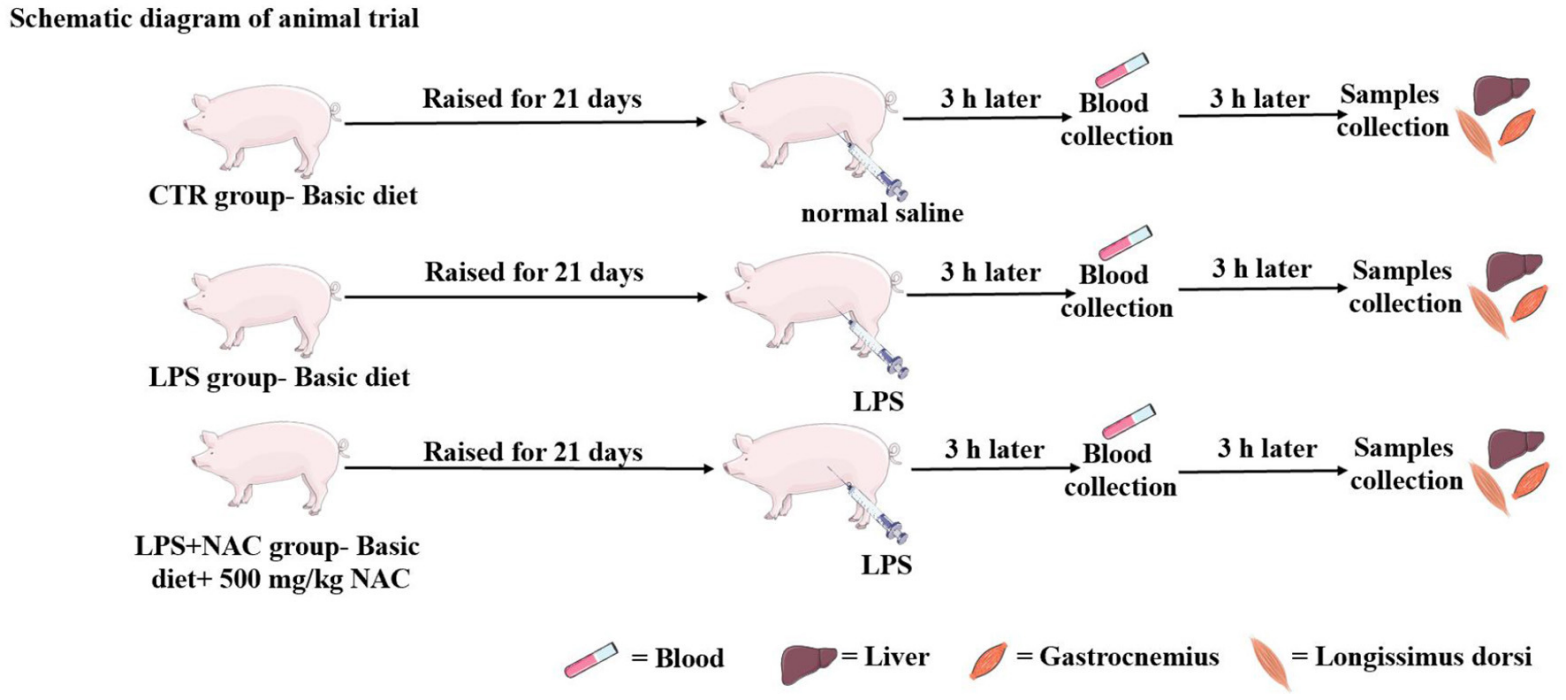
The operation progress of animal trial. The piglets were raised for 21 days, the LPS and normal saline were injected intraperitoneally at the end of feeding trial. Three hours later, the blood was harvested from the jugular vein, and then the liver and muscle were collected 6 h later.

The animal experiment was carried out at Wuhan Polytechnic University, and the animal use protocol was approved by the Animal Care and Use Committee of Hubei Province, and the animal welfare number was WPU202005001. Twenty-four healthy male crossbred piglets of the same genetic background (Durc × Landrace × Yorkshire) were weaned at 21 ± 2 days of age and then scheduled for a 3-day acclimation period. Afterwards, all piglets were weighed and assigned to three treatment groups according to uniform average body weights of each treatment group (BW, 5.03 ± 0.59 kg). Piglets in the control and LPS groups were fed a basal diet formulated to meet the nutritional requirements of piglets as described in the National Research Council (NRC- 2012). The formulation and nutrient levels of the diets are described in Table 1. The diet of LPS+ NAC group was supplemented with 500 mg/kg NAC (Sigma Chemical Inc., St. Louis, MO, USA, purity > 99%) in the basal diet. The feeding trial lasted for 21 days. At the end of the trial, piglets in the LPS and LPS+ NAC groups were injected intraperitoneally with LPS (*E. coli* serotype 055: B5; Sigma Chemical Inc., St. Louis, MO, USA) at the dose of 100 μg/kg BW and the control group received the same volume of normal saline. Blood was collected from the jugular vein 3 h later, and all pigs were anesthetized by intramuscular injection of sodium pentobarbital (50 mg/kg BW) 6 h after LPS or saline injection. All piglets were then slaughtered and muscle and liver samples were harvested within 15 min following the protocol described by Hou et al. (13). Briefly, the abdominal cavity of the anesthetized and euthanized piglets was opened, the liver was harvested and the tip of the right hepatic lobule was collected. A ~2 cm^2^ longissimus dorsi was obtained from the left side of the pig at 5 cm of the dorsal midline of the 3rd to 4th reciprocal rib spacing. A ~2 cm^2^ gastrocnemius was collected from the dorsal end of the lower end of the femur to the apex of the calcaneus. The liver and muscles were collected and cut up with scissors on the ice, frozen in liquid nitrogen, and transferred to the refrigerator for storage at −80°C. The roadmap of the trial is depicted in Figure 1. During the feeding trial, all piglets were housed individually in stainless steel metabolic cages (1.20 × 1.10 m^2^) with free access to water and food. The room temperature was maintained at 25 ± 2°C, and q 24-hour lighting schedule was implemented.

**Table 1:** Dietary formulations and nutrient levels of basal diets (airdry basis).

**Ingredients**	**Content %**		
Corn	61.88	Nutrient levels^b^	
Soybean meal	21.98	Digestible energy, MJ/kg	14.22
Wheat middlings	4.00	Crude protein, %	20.90
Whey powder	3.00	Threonine, %	0.74
Fish meal	3.00	Methionine, %	0.30
Soybean protein concentrate	1.50	Methionine+ Cystine, %	0.65
CaHPO_4_	1.25	Lysine, %	1.15
Premix^a^	1.00	Tryptophan, %	0.21
Limestone	0.69	Calcium, %	0.70
NaCl	0.30	Total phosphorus, %	0.60
Acidifier	0.30	Available phosphorus, %	0.32
Soybean oil	0.50		
Lysine hydrochloride	0.25		
Choline chloride	0.20		
Mold inhibitor	0.10		
DL-methionine	0.05		
Total	100.00		

^a^The premix provided the following per kg of diet, VA = 10,800 IU, VD_3_ = 4,000 IU, VE = 40 IU, VK_3_ = 4 mg, VB_1_ = 6 mg, VB_2_ = 12 mg, VB_6_ = 6 mg, VB_12_ = 0.05 mg, Biotin = 0.2 mg, Folic acid = 2 mg, Nicotinic acid = 50 mg, D-calcium pantothenatc = 25 mg, Fe (as ferrous sulfate) = 100 mg, Cu (as copper sulfate) = 150 mg, Mn (as manganese sulfate) = 40 mg, Zn (as zinc sulfate) = 100 mg, I (as potassium iodide) = 0.5 mg, and Se (as sodium selenite) = 0.3 mg.

^b^Digestible energy was a calculated value and others were measured values.

### 2.2 Blood biochemical parameters and hormones

Blood was collected from the jugular vein and analyzed using an automated blood cell analyzer (Siemens ADVIA^®^ 2120i, Germany) to determine the white blood cell count and, the percentage of neutrophils, lymphocytes, and monocytes. The plasma was obtained by centrifuging the blood at 3,500 × *g* and 4°C for 10 min and was stored at −80°C for further use. The levels of γ-glutamyl transferase (GGT) and glucose (GLU) was measured using an automated biochemical analyzer (HITACHI 7020, Japan). The levels of glucagon, epidermal growth factor (EGF), and insulin C-peptide (C-P) were measured using radio-immunoassay kits purchased from Beijing SINO-UK Institute of Biological Technology, Beijing, China, with a detection coefficient of variation of < 10%.

### 2.3 Growth-related parameters, antioxidant enzymes, and oxidative products

0.1 g of frozen liver and muscle tissue were weighed and placed into a 2 mL clean RNA-free polystyrene tube. Then, 900 μL of pre-cooled normal saline was added and mixed evenly on ice with a homogenizer until no obvious graininess was present. The mixture was then centrifuged at 3,500 × *g* and 4°C for 15 min, and the supernatant was separated and frozen at −20°C for later use. TRI reagent-DNA and RNA isolation reagent were used to extract DNA and RNA from the longissimus dorsi, gastrocnemius and liver. A colorimetric method was used to determine their concentrations (14), using a kit purchased from Nanjing Jiancheng Institute of Biological Engineering, Jiangsu, China. Total protein (TP) levels in the longissimus dorsi, gastrocnemius, and liver were measured using kits from the same manufacturer. The ratios of RNA to DNA and TP to DNA were calculated. The levels of glutathione peroxidase (GSH-px), total superoxide dismutase (T-SOD), catalase (CAT), H_2_O_2_, and malondialdehyde (MDA) in the longissimus dorsi and gastrocnemius were measured according to the protocol of kits purchased from Nanjing Jiancheng Institute of Biological Engineering, Jiangsu, China.

### 2.4 The levels of IL-1β, IGF-1, and PGE-2 in the liver

The liver tissue homogenate was prepared using the method described above. The levels of insulin-like growth factor 1 (IGF-1) and prostaglandin E2 (PGE-2) in the liver were then analyzed using commercially available ^125^I kits purchased from the Beijing SINO-UK Institute of Biological Technology, Beijing, China. The coefficient of variation for the detection of IGF-1 and PGE-2 was < 15%. The content of IL-1β in the liver was measured using an ELISA kit purchased from RD Systems, Quantikine, USA. The level of total protein in the liver was determined according to the protocol of kits purchased from Nanjing Jiancheng Institute of Biological Engineering, Jiangsu, China. And then, the levels of IL-1β, IGF-1, and PGE-2 were presented based on the measured levels divided by the total protein.

### 2.5 The levels of total GSH and cysteine in the liver and muscle

The sample preparation and pre-treatment method described by Yi et al. (12) was used, and the levels of total GSH and cysteine were measured using the high-performance liquid chromatography (HPLC) method described in a previous study (15). Briefly, 0.05 g of frozen longissimus dorsi, gastrocnemius and liver were homogenized with 1.5 mL of homogenization buffer. The mixture was then transferred into a clean 15 mL polystyrene tube, and 0.75 mL of 2 M K_2_CO_3_ was added and mixed well. The mixture was centrifuged at 3,000 × *g* for 5 min, and 50 μL of the supernatant was harvested and stored at −20°C before derivatization with 25 mM iodoacetic acid. The HPLC system used a model 2475 multi-λ fluorescence detector, a supelco C18 guard column (4.6 mm × 5 cm, 20–40 μm, Sigma-Aldrich, St. Louis, MO, USA), and a supelco C18 column (4.6 mm × 15 cm, 3 μm, Sigma-Aldrich, St. Louis, MO, USA). The system also required a 717 plus WISP Autosampler and a 600E powerline multisolvent delivery system equipped with 100 μL heads. The level of total protein in the liver and muscles were determined according to the protocol of kits purchased from Nanjing Jiancheng Institute of Biological Engineering, Jiangsu, China. Finally, the sample detection and data analysis followed the protocol described by Yi et al. (16).

### 2.6 Detection and analysis of gene mRNA levels

The longissimus dorsi, gastrocnemius and liver were harvested and immediately frozen in the liquid nitrogen. 0.1 g samples were added to 1 mL of TRIzol reagent (Invitrogen, Carlsbad, CA, USA) in a 1.5 mL clean, RNA-free polystyrene tube, Total RNA was extracted and quality control was performed according to the method described by Hou et al. (17). The extracted RNA was reverse-transcribed using the PrimeScript^®^RT reagent kit with gDNA Eraser (Takara, Dalian, China), and the resulting cDNA was stored at −80°C for later use. The RT-PCR reaction was performed using an Applied Biosystems 7500 Fast Real-Time PCR System (Foster City, CA, USA). The necessary materials for this process included cDNA, SYBR^®^ Premix Ex Taq™ (Takara, Dalian, China), and gene-specific primers listed in Table 2. The RT-PCR procedure described by Yi et al. (12) was used in this study. To ensure reliable results, the glyceraldehyde-3-phosphate dehydrogenase (GAPDH) was used as internal reference gene for liver, ribosomal protein L4 (RPL4) and hypoxanthine-guanine phosphoribosyl transferase 1 (HPRT1) were used as internal reference genes for longissimus dorsi and gastrocnemius, respectively. Data were analyzed using the 2^−Δ*ΔCt*^ method (18), and all samples were run in triplicate.

**Table 2:** The primer sequences of the genes.

**Gene name**	**Forward primer, 5^′^-3^′^**	**Reversed primer, 5^′^-3^′^**
*HIF-1*	TTACTCATCCgTgCgACCAT	CTCCgCTgTgTATTTTgCTCTTT
*Nrf-2*	gAAgTgATCCCCTgATgTTgC	ATgCCTTCTCTTTCCCCTATTTCT
*γ-GCS*	CgTTCACACTCTTCCCCTCACT	AgTTgTCCCTTTTgATggTgCT
*GSH-Px*	ACAACggTgCgggACTACA	CgCCATTCACCTCACACTTC
*mTOR*	TTgTTgCCCCCTATTgTgAAg	CCTTTCgAgATggCAATggA
*P70S6K*	ggAAACAAgTggAATAgAgCAgATg	TTggAAgTggTgCAgAAgCTT
*4EBP1*	CCggAAgTTCCTAATggAgTgT	ggTTCTggCTggCATCTgT
*PGC-1α*	gATgTgTCgCCTTCTTgTTC	CATCCTTTggggTCTTTgAg
*CAST*	TCCAAgTCAggAgAACAgAAAgg	TgAAgCAgAggAAggCgATAC
*HFABP*	CgCCTgTTCTgTCgTCTCTTT	TCTTgCTgTCCACTAgCTTCCA
*EGFR*	ggCCTCCATgCTTTTgAgAA	gACgCTATgTCCAggCCA A
*IGF1*	gCCCAAggCTCAgAAgg	TTTAACAggTAACTCgTgC
*IGF1R*	ATTCgCACCAATgCTTCA	AgggCgggTTCCACTTC
*IFN-β*	AgCAgATCTTCggCATTCTC	gTCATCCATCTgCCCATCAA
*MX1*	AgTgCggCTgTTTACCAAg	TTCACAAACCCTggCAACTC
*OAS1*	TggTggTggAgACACACACA	CCAACCAgAgACCCATCCA
*OASL*	ggCACCCCTgTTTTCCTCT	AgCACCgCTTTTggATgg
*IFIT1*	gCTAAACCAAACACCgCAgAA	ggAACTCAATCTCCTCCAAgACC
*IFIT3*	gCATTTTCCAgCCAgCATC	TCTgTTCCTTTCCTTTCCTTCCT
*IFIT5*	CAgAAAATACAgCCATCCACCA	AgggCACTTAAACTCTgCACATC
*CXCR3*	TgTAgCCAAgAAAgTAgggTggA	AggCgTAgAgCAgTgggTTg
*HSPB1*	ATgAgCACggCTTCATTTCC	gggCTTTTCCgACTTTCCA
*IFN-γ*	TCTgggAAACTgAATgACTTCg	gACTTCTCTTCCgCTTTCTTAggTT
*GAPDH*	CgTCCCTgAgACACgATggT	CCCgATgCggCCAAAT
*RPL4*	gAgAAACCgTCgCCgAAT	gCCCACCAggAgCAAgTT
*HRPT1*	AACCTTgCTTTCCTTggTCA	TCAAgggCATAgCCTACCAC

HIF-1, hypoxia inducible factor-1; Nrf-2, nuclear factor erythroid 2-related factor 2; γ-GCS, γ-glutamylcysteine synthetase; GSH-Px, glutathione peroxidase; mTOR, mechanistic target of rapamycin; P70S6K, p70 ribosomal protein S6 kinase; 4EBP1, 4E binding protein 1; PGC-1α, peroxisome proliferators-activated receptor γ coactivator 1 alpha; CAST, calpastatin; HFABP, heart fatty acid binding protein; EGFR, epidermal growth factor receptor; IGF-1/1R, insulin like growth factor 1/1 receptor; IFN-β/γ, interferon-β/γ; MX1, myxovirus resistance 1; OAS1/L, 2′; 5′- oligadenylate synthase 1/L; IFIT1/3/5, interferon-induced protein with tetratricopeptide repeats 1/3/5; CXCR3, chemokine (C-X-C motif) receptor 3; HSPB1, heat-shock protein beta-1; GAPDH, glyceraldehyde-3-phosphate dehydrogenase; RPL4, ribosomal protein L4; HRPT1, hypoxanthine-guanine phosphoribosyl transferase 1.

### 2.7 Data analysis

The data was analyzed using the one-way ANOVA program in SPSS 23.0 software (SPSS Inc., Chicago, IL), followed by Duncan's multiple comparisons to investigate the differences between groups. And the independent sample *T*-test was used to analyze the differences between Control and LPS group, between LPS and LPS+NAC group. Pearson correlation analysis was conducted to examine correlations between certain indicators. All data were presented as means± standard deviation, and a *P*-values < 0.05 was considered statistically significant, 0.05 < *P*-values < 0.1 indicated a trend of significant differences between groups additionally. Graphpad prism 8.0 software was utilized for data visualization.

## 3 Results

### 3.1 Blood indices and hormones

The percentage of monocytes was decreased with LPS challenge (*P* < 0.05; Table 3), and the level of GGT in the plasma tended to increase in the LPS group (*P* = 0.095). Additionally, the content of glucagon in the plasma increased and the EGF decreased with LPS treated. Compared to the LPS group, dietary supplementation of 500 mg/kg NAC down-regulated the level of glucagon in the serum (*P* < 0.05) and elevated the content of EGF (*P* = 0.089).

**Table 3:** The results of blood related parameters.

**Items**	**Control**	**LPS**	**LPS+ NAC**	***P*-value**
**Blood cells and biochemical parameters**				
WBC, 10 e^3^/uL	11.80 ± 2.76	13.43 ± 2.67	10.15 ± 2.40	0.129
NEU, %	47.71 ± 9.48	55.38 ± 3.47	52.90 ± 8.63	0.222
LYM, %	32.00 ± 6.54	34.55 ± 10.58	34.00 ± 10.39	0.882
MONO, %	13.90 ± 3.35^a^	8.21 ± 3.06^b^	10.98 ± 3.80^ab^	0.037
GLU, mmol/L	5.81 ± 0.37	5.09 ± 0.64	5.05 ± 1.55	0.356
GGT, U/L	33.50 ± 4.14	38.97 ± 6.75	40.70 ± 6.56	0.095
**Hormones**				
Glucagon, pg/ml	134.51 ± 36.20^c^	273.49 ± 76.53^a^	204.32 ± 34.98^b^	0.001
C-P, ng/ml	0.21 ± 0.06	0.20 ± 0.04	0.22 ± 0.05	0.854
EGF, ng/ml	1.14 ± 0.36	0.79 ± 0.09	1.06 ± 0.27	0.083

^a, b, c^Means in the same row without common superscripts differ significantly (P < 0.05). Data were presented as mean ± standard deviation.

WBC, white blood cells; NEU, neutrophile; LYM, lymphocyte; MONO, monocyte; GLU, glucose; GGT, gamma-glutamyl transpeptidase; C-P, insulin C-peptide; EGF, epidermal growth factor.

### 3.2 Antioxidant enzymes and oxidative products, developmental ability of muscles

The levels of H_2_O_2_ and MDA were increased in the longissimus dorsi and gastrocnemius muscles, and the content of CAT in the gastrocnemius was decreased with LPS treated (*P* < 0.05; Table 4). However, dietary supplementation with NAC was able to alleviate these effects. Furthermore, compared to the LPS group, the level of GSH-px in the longissimus dorsi and gastrocnemius muscles increased with NAC supplementation (*P* < 0.05). Additionally, the levels of DNA and the ratio of RNA to DNA in the longissimus dorsi, and the level of DNA in the gastrocnemius were decreased with LPS challenge (*P* < 0.05; Table 5). Interestingly, the addition of NAC to the diet also alleviated these effects.

**Table 4:** The levels of Antioxidant enzymes and peroxide products in the muscle.

**Items**	**Control**	**LPS**	**LPS+ NAC**	***P*-value**
**Longissimus dorsi**				
GSH-Px, U/mg prot	64.58 ± 9.43^ab^	56.64 ± 9.92^b^	71.27 ± 10.95^a^	0.029
T-SOD, U/mg prot	90.58 ± 6.42	85.14 ± 10.43	85.50 ± 6.48	0.333
CAT, U/mg prot	5.28 ± 1.00^a^	4.87 ± 0.65^ab^	4.16 ± 0.53^b^	0.023
H_2_O_2_, mmol/mg prot	62.46 ± 4.22^b^	72.52 ± 10.52^a^	61.41 ± 9.33^b^	0.029
MDA, nmol/mg prot	0.29 ± 0.06^b^	0.45 ± 0.09^a^	0.35 ± 0.10^b^	0.006
**Gastrocnemius**				
GSH-Px, U/mg prot	52.67 ± 10.32^b^	53.83 ± 12.43^b^	85.28 ± 7.36^a^	< 0.001
T-SOD, U/mg prot	62.81 ± 6.36^a^	56.12 ± 8.07^ab^	54.91 ± 4.52^b^	0.050
CAT, U/mg prot	1.73 ± 0.23^b^	1.32 ± 0.27^c^	2.10 ± 0.33^a^	< 0.001
H_2_O_2_, mmol/mg prot	41.26 ± 4.80^c^	64.07 ± 8.98^a^	53.77 ± 7.39^b^	< 0.001
MDA, nmol/mg prot	4.23 ± 0.87^b^	5.27 ± 0.90^a^	5.55 ± 0.59^a^	0.008

^a, b, c^Means in the same row without common superscripts differ significantly (P < 0.05). Data were presented as mean ± standard deviation.

GSH-Px, glutathione peroxidase; T-SOD, total superoxide dismutase; CAT, catalase; MDA, malondialdehyde; prot, protein.

**Table 5:** The levels of growth-related indicators in the muscle.

**Items**	**Control**	**LPS**	**LPS+ NAC**	***P*-value**
**Longissimus dorsi**				
DNA, μg/mg protein	0.32 ± 0.07^a^	0.24 ± 0.05^b^	0.30 ± 0.05^a^	0.023
RNA/DNA	0.71 ± 0.15^a^	0.57 ± 0.10^b^	0.73 ± 0.09^a^	0.020
Protein/DNA	263.32 ± 68.71	235.14 ± 50.05	246.26 ± 26.71	0.553
**Gastrocnemius**				
DNA, μg/mg protein	0.55 ± 0.04^a^	0.49 ± 0.03^b^	0.58 ± 0.08^a^	0.006
RNA/DNA	0.28 ± 0.03	0.30 ± 0.02	0.31 ± 0.04	0.134
Protein/DNA	313.75 ± 26.84	289.67 ± 52.14	304.84 ± 41.45	0.513

^a, b^Means in the same row without common superscripts differ significantly (P < 0.05). Data were presented as mean ± standard deviation.

### 3.3 Contents of total GSH and cysteine as well as the genes transcription levels in the longissimus dorsi and gastrocnemius

**Figure 2:**
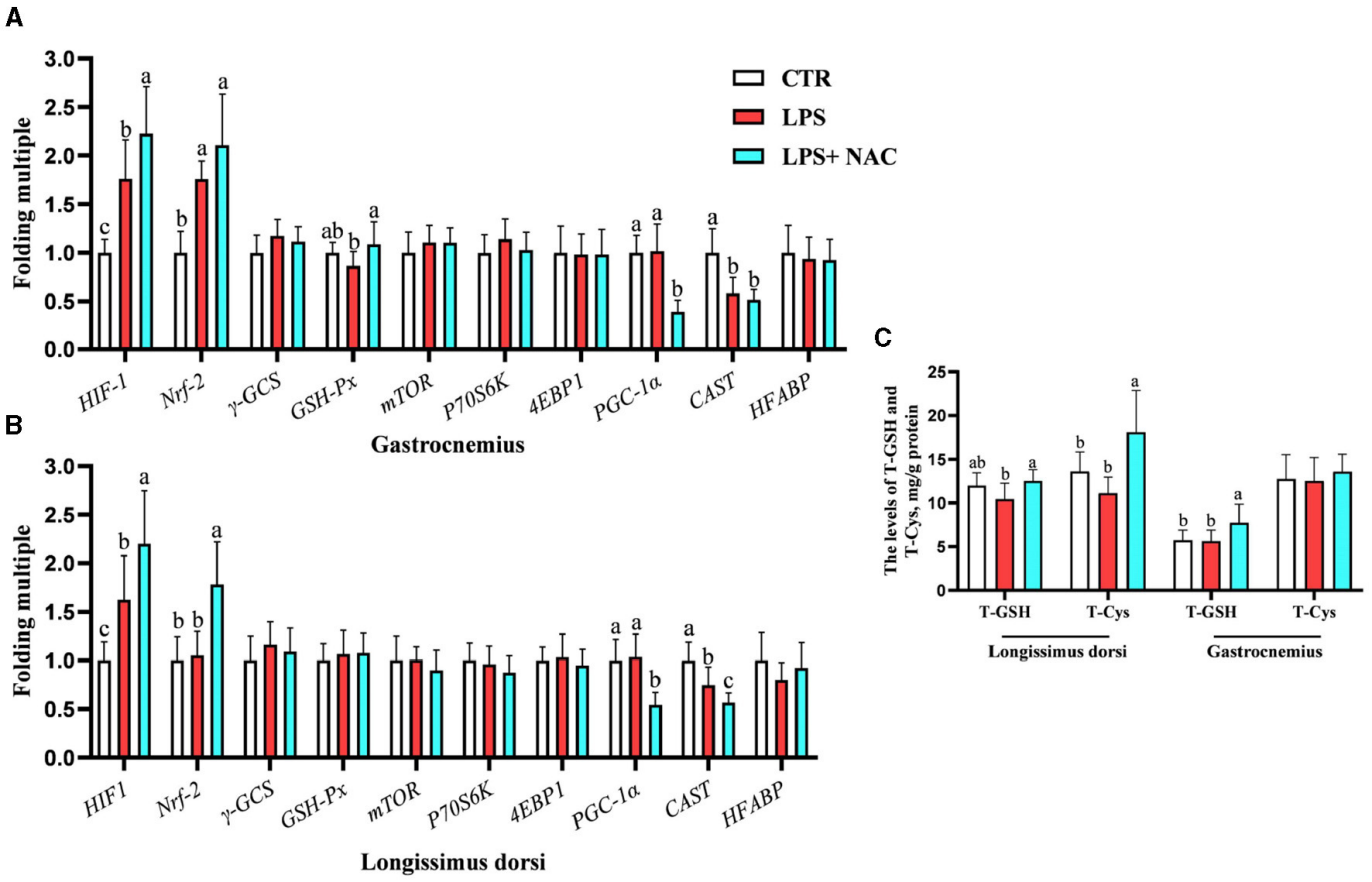
The transcription levels of some genes and the levels of total GSH and cysteine in the muscle. Among them, the transcription levels of some genes in gastrocnemius and longissimus dorsi were arranged in **(A, B)**, respectively. The levels of total GSH and cysteine in the muscle were showed in **(C)**. ^a, b, c^Means in the same row without common superscripts differ significantly (*P* < 0.05). Data were presented as mean ± standard deviation. The full names of genes refer to the remarks in Table 2. T-GSH, total glutathione peroxidase; T-Cys, total cysteine.

Compared to the LPS group, dietary supplementation of NAC increased the levels of total GSH and cysteine in the longissimus dorsi and the content of total GSH in the gastrocnemius (*P* < 0.05; Figure 2C). The genetic outcomes indicated that the mRNA levels of hypoxia-inducible factor-1 (*HIF-1*) and nuclear factor erythroid 2-related factor 2 (*Nrf-2*) in the gastrocnemius and *HIF-1* in the longissimus dorsi were up-regulated with LPS treatment (*P* < 0.05; Figures 2A, B). The transcription level of calpastatin (*CAST*) in the longissimus dorsi and gastrocnemius was down-regulated with LPS challenge (*P* < 0.05). Compared to the LPS group, the mRNA levels *HIF-1, Nrf-2*, and glutathione peroxidase (*GSH-px*) in the gastrocnemius and *HIF-1* and *Nrf-2* in the longissimus dorsi were up-regulated with NAC treatment in the diet. Additionally, dietary supplementation of NAC down-regulated the transcription levels of peroxisome proliferators-activated receptor γ coactivator 1 alpha (*PGC-1*α) in the longissimus dorsi and gastrocnemius (*P* < 0.05).

**Figure 3:**
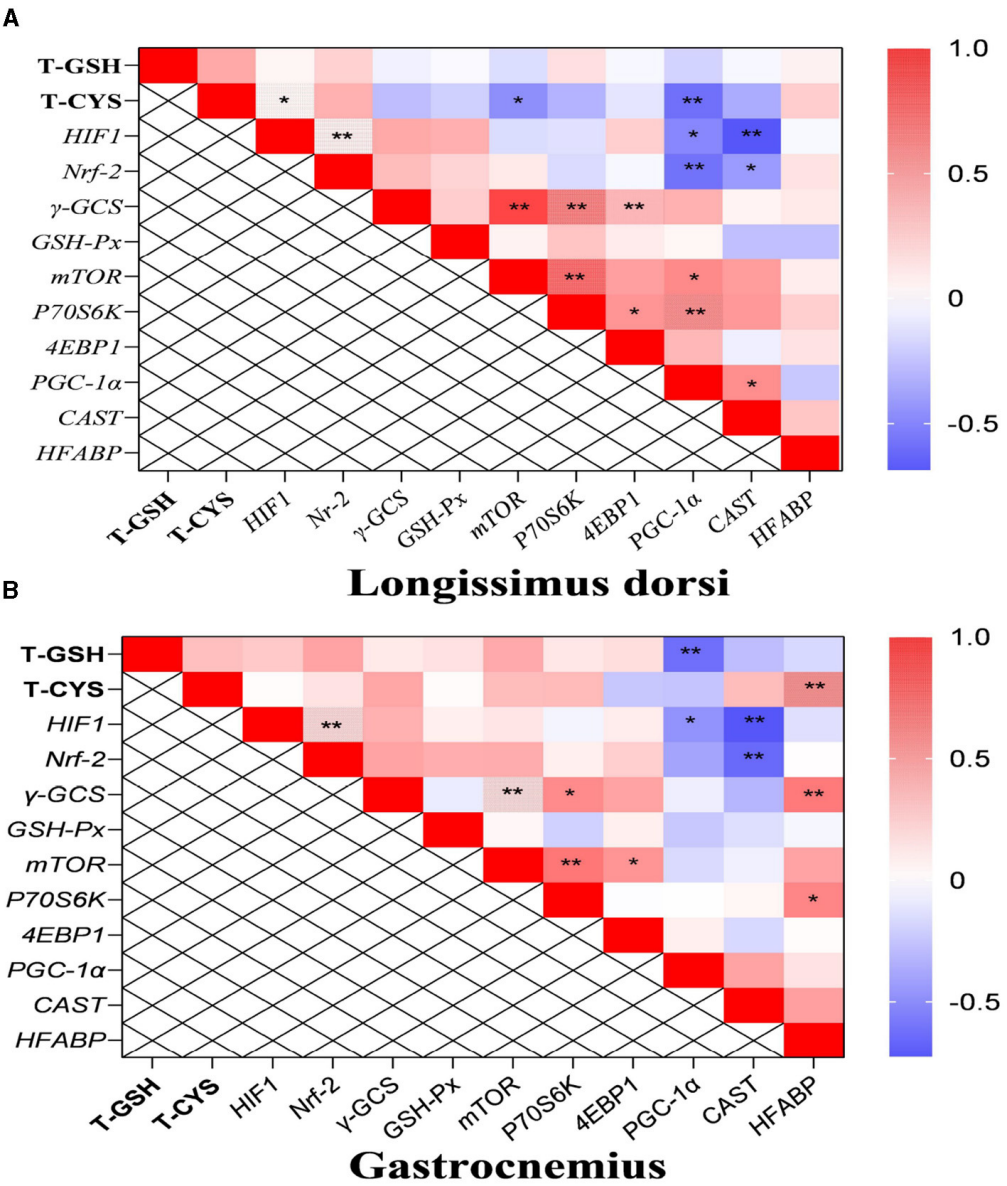
The results of correlation analysis between some indicators in muscle. Among them, the results of gastrocnemius and longissimus dorsi were arranged in **(A, B)**, respectively. The level of Pearson correlation coefficient was used to make a heat map, *Means the differences were significant (0.01 < *P* < 0.05), ** was regarded as the differences were extremely significant (*P* < 0.01). Additionally, the red color indicate a strong positive correlation (correlation coefficient = 1), while the purple color indicates a strong negative correlation (correlation coefficient = −1). The full names of genes refer to the remarks in Table 2 and Figure 2.

In the longissimus dorsi, the correlation analysis results showed that the level of total cysteine was positively correlated with the transcription level of *HIF-1*, and the mRNA level of *HIF-1* was also positively correlated with that of *Nrf-2* (*P* < 0.05; Figure 3A). Additionally, the transcription level of *PGC-1*α was negatively correlated with the content of total cysteine and the mRNA levels of *HIF-1* and *Nrf-2*. The mRNA level of *CAST* was also negatively correlated with the transcription levels of *HIF-1* and *Nrf-2*. In the gastrocnemius, the mRNA levels of *PGC-1*α and *CAST* were negatively correlated with the transcription level of *HIF-1* (*P* < 0.05; Figure 3B).

### 3.4 The transcription levels of the genes, the contents of total GSH and cysteine, as well as the immune and growth-related indicators in the liver

**Figure 4:**
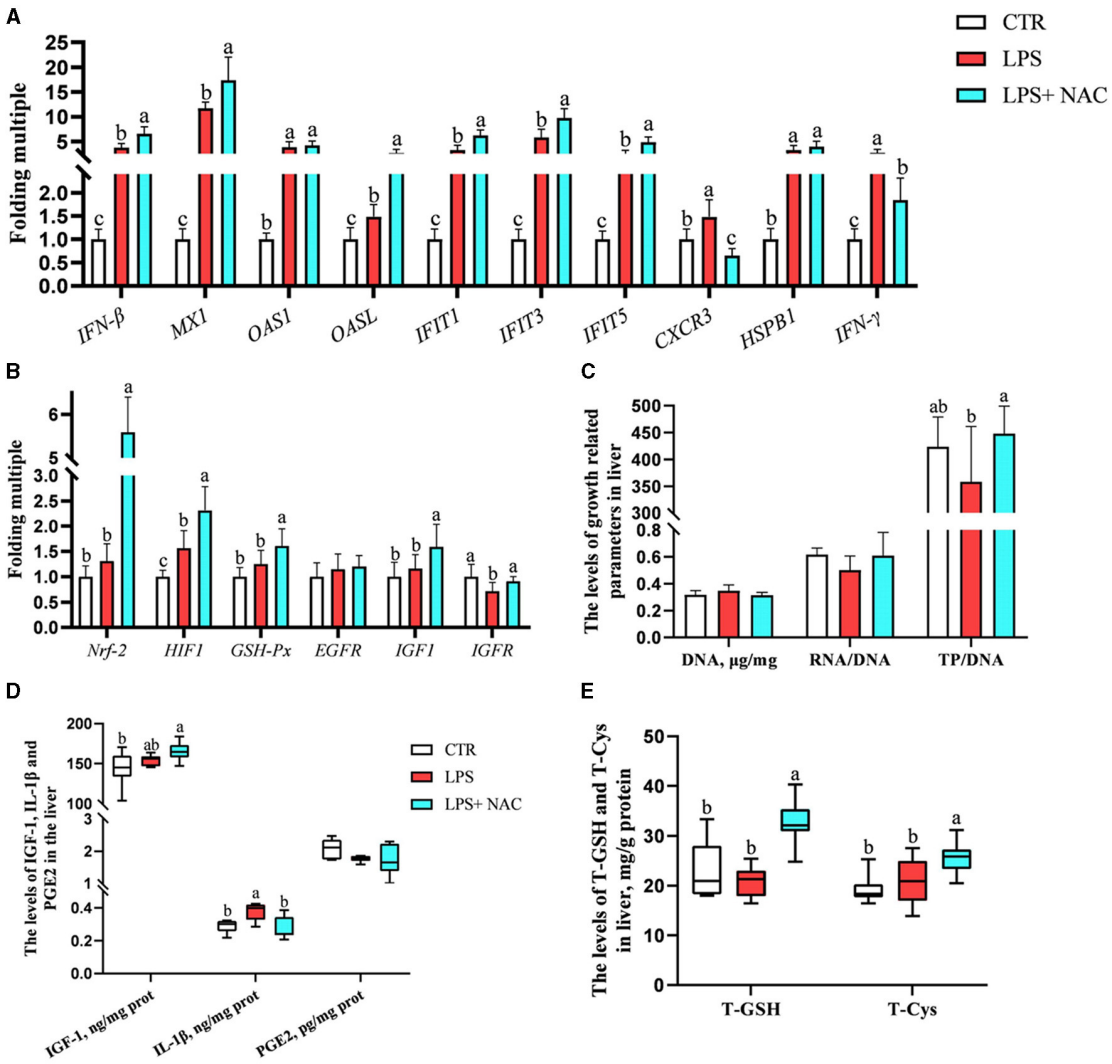
The transcription levels of some genes, the levels of total GSH and cysteine, and the immune and growth-related indicators in the liver. Among them, the transcription levels of some genes were arranged in **(A–C)** showed the levels of DNA, the ratio of RNA to DNA, and the ratio of TP to DNA. Additionally, the contents of IGF-1, IL-1β, and PGE-2 were arranged in **(D)**, and the levels of total GSH and cysteine were showed in **(E)**. ^a, b, c^Means in the same row without common superscripts differ significantly (*P* < 0.05). Data were presented as mean ± standard deviation. The full names of genes refer to the remarks in Table 2. T-GSH, total glutathione peroxidase; T-Cys, total cysteine; IGF-1, insulin like growth factor 1; IL-1β, interleukin-1β; PGE-2, prostaglandin E-2; prot, protein.

The mRNA levels of interferon-β/γ (*IFN-*β, *IFN-*γ), myxovirus resistance-1 (*MX-1*), 2′, 5′- oligadenylate synthase 1/L (*OAS1, OASL*), interferon-induced protein with tetratricopeptide repeats 1/3/5 (*IFIT1, IFIT3, IFIT5*), chemokine (C-X-C motif) receptor 3 (*CXCR3*), heat-shock protein beta-1 (*HSPB-1*), and *HIF-1* were up-regulated in the liver, while the transcription level of insulin like growth factor receptor (IGF*-R*) was down-regulated following LPS challenge (Figures 4A, B). Compared to the LPS group, dietary supplementation of NAC elevated the mRNA levels of *IFN-*β, *MX-1, OASL, IFIT1, IFIT3, IFIT5, Nrf-2, HIF-1, GSH-Px, IGF-1*, and IGF*-R* in the liver. Additionally, the up-regulated mRNA levels of *CXCR3* and *IFN-*γ in the LPS group were down-regulated with NAC treatment. The ratio of total protein to DNA in the liver showed a tendency to decrease in the LPS group (*P* = 0.074), but dietary supplementation with NAC was able to alleviate this effect (*P* < 0.05; Figure 4C). The content of IL-1β in the liver increased with LPS treatment, and dietary supplementation with NAC also alleviated this effect (*P* < 0.05; Figure 4D). Compared to the LPS group, the levels of IGF-1 (*P* = 0.058), total GSH and cysteine (*P* < 0.05) were increased (Figure 4E).

**Figure 5:**
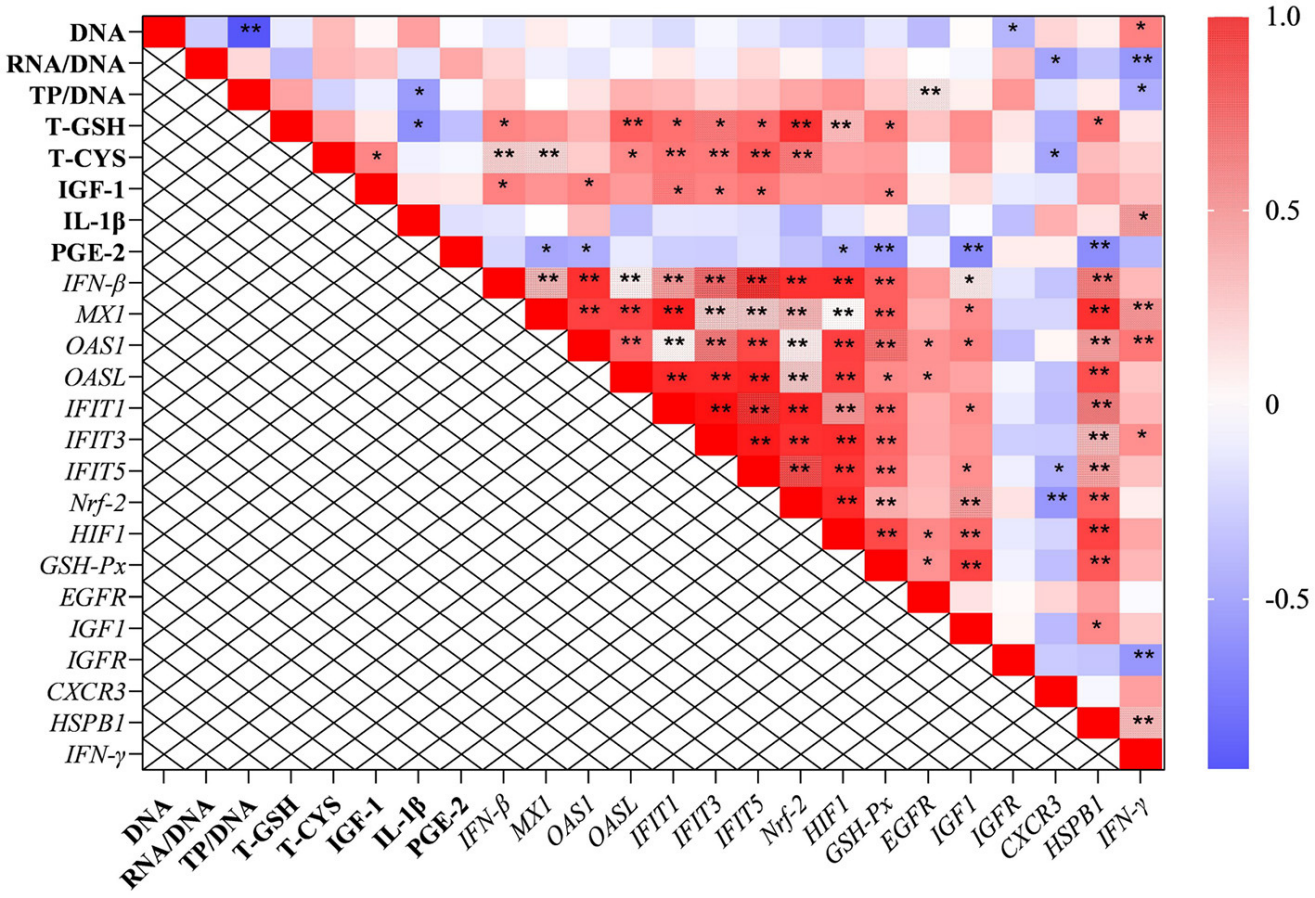
The results of correlation analysis between some indicators in the liver. Among them, the level of Pearson correlation coefficient was used to make a heat map, * means the differences were significant (0.01 < *P* < 0.05), ** was regarded as the differences were extremely significant (*P* < 0.01). Additionally, the red color indicate a strong positive correlation (correlation coefficient= 1), while the purple color indicates a strong negative correlation (correlation coefficient = −1). The full names of genes refer to the remarks in Table 2 and Figures 2–4.

The correlation analysis results suggested that the level of total cysteine was positively correlated with IGF*-1* and negatively correlated with the mRNA level of *CXCR3* in the liver. The content of total GSH was negatively correlated with IL-1β (*P* < 0.05; Figure 5). Additionally, the ratio of total protein to DNA was negatively correlated with the level of IL-1β and the transcription level of *IFN-*γ. It was worth mentioning that the transcription levels of type I interferon-related genes were positively correlated with the levels of IGF-1, total cysteine, and GSH.

## 4 Discussion

Under normal conditions, the body's antioxidant and oxidative systems are in a dynamic balance. However, when the oxidative system became dominant, some oxygen radicals and peroxidation products, such as H_2_O_2_ and malondialdehyde, were produced, which could lead to tissue injury (15). To counteract this, the antioxidant system was activated and certain antioxidant enzymes, such as SOD, CAT and GSH, were released to mitigate the effects (19, 20). Studies have shown that LPS challenge could elevate peroxidation product levels and decrease antioxidant enzyme levels, leading to tissue oxidative stress (21). In the present study, we observed the consistent outcomes. NAC, a widely accepted antioxidant, is a prerequisite for cysteine synthesis. In our previous studies, it has been demonstrated that NAC is useful for antioxidant function in piglet intestines (7). In this study, dietary supplementation of NAC increased total cysteine and GSH levels and promoted the expression of antioxidant enzymes while inhibiting peroxidation product production, thereby alleviating the adverse effects of LPS on piglet muscle.

The beneficial effect of NAC on the antioxidant function of piglets is undeniable. And the signaling pathway related to Nrf-2 is mainly involved in the regulation of antioxidant capacity. Notably, the Nrf-2 signaling pathway is considered to be the dominant classical pathway in the antioxidant system that was activated to promote the expression of antioxidant enzymes, for instance, GSH-Px, SOD, and CAT, and thereby alleviated oxidative stress (22). We hypothesized that the improvement in antioxidant capacity of NAC could be attributed to this. To further support this idea, certain genes about Nrf-2 signaling pathway were investigated. The outcomes showed that NAC contributed to the up-regulation of the transcription levels of *Nrf-2, GSH-Px*, and *HIF-1*. Study demonstrated that HIF-1 played an important role in maintaining the balance between the body's oxidative and antioxidant systems (23). *Nrf-2* and *HIF-1* were elevated with NAC treated, accordingly, the up-regulation of antioxidant enzymes and the down-regulation of peroxide products were observed in the present study. This might be one of the mechanisms by which NAC alleviated oxidative stress induced by LPS in piglets. However, with NAC treated, how the activation of Nrf-2 was further related to HIF-1 might need to be investigated in the future. Although a detailed investigation of it was beyond the scope of this work, our findings revealed that the beneficial effect of NAC on the antioxidant capacity of piglets could be modulated by activating the Nrf-2/HIF-1 signaling pathway.

Oxidative stress caused tissue injury and negatively affected the meat quality (24). Hou et al. (14) suggested that DNA and RNA levels and the ratio of total protein to DNA could be used to assess the developmental ability of tissue production. In the present study, DNA levels and the ratio of total protein to DNA decreased with LPS treatment, but dietary supplementation of NAC was able to alleviate this effect. It demonstrated that NAC was helpful for the growth of muscle challenged with LPS, which was generally consistent with our previous study (12). To investigate the mechanisms involved, we pay our attention to the mTOR and EGF signaling pathway, On account of it was involved in the regulation of muscle growth and development has been intensively investigated(13, 25). Studies demonstrated that the mTOR signaling pathway regulated protein synthesis and muscle growth, and functional amino acids, such as arginine and NAC, contributed to tissue growth and development by activating the mTOR signaling pathway (12, 13). In the present study, dietary supplementation of NAC failed to up-regulate the transcriptional levels of genes related to the mTOR signaling pathway, which was inconsistent with previous studies (12, 13). A possible explanation was that the effect of NAC on the mTOR signaling pathway might be more characterized in the intestine. Additionally, different genetic backgrounds of piglets and feeding conditions might also be factors. Nevertheless, we still believed that NAC was useful for muscle growth due to the observed up-regulation of EGF in the blood. Studies demonstrated that EGF contributed to cell and tissue growth and the maintenance of immune homeostasis (25, 26). Dietary supplementation of NAC might alleviate muscle injury treated with LPS by promoting the expression of EGF rather than by activating the mTOR signaling pathway in the present study.

The tenderness of meat is a matter of concern for consumers as it affects the taste. Multiple genes regulate tenderness. A previous study suggested that PGC-1α was positively correlated with the level of muscle fiber, and its high expression would worsen the tenderness of meat (27). Another study demonstrated that *CAST* also played a role in regulating meat tenderness (28). In this study, the mRNA level of *CAST* was down-regulated in the longissimus dorsi and gastrocnemius with LPS challenge, indicating a negative effect on meat tenderness. Intriguingly, dietary supplementation of NAC down-regulated the transcriptional level of *PGC-1*α in these muscles. Correlation analysis revealed that the mRNA level of *PGC-1*α was negatively correlated with the contents of total GSH and cysteine, as well as the transcriptional levels of *Nrf-2* and *HIF-1*. Additionally, NAC contributed to muscle growth and development due to its regulatory role in the EGF signaling pathway as mentioned above. These results suggested that NAC might improve the meat quality by regulating multiple targets, such as, Nrf-2/HIF-1 and EGF signaling pathways.

A study demonstrated that LPS caused inflammation by activating the TLR4-NF-κB signaling pathway, which weakened the body's innate immunity (29). It is well-established that the mononuclear macrophage system is involved in mediating innate immune function. In the present study, the percentage of monocytes in the blood was reduced with LPS treatment. This provided additional evidence that LPS caused innate immunosuppression in piglets. In inflammatory states, the levels of some hormones were disturbed, such as the up-regulation of glucagon (30). In this study, the glucagon level was elevated by LPS treatment. To account for this effect, we may hypothesize that in response to the LPS challenge, a significant amount of glycogen was broken down, leading to the mobilization of glucagon to regulate blood glucose levels. However, the inflammatory response induced by LPS might lead to negative impacts on tissues, hence we observed that the level of GGT in the serum was elevated with LPS challenge. Results also showed that the level of IL-1β was elevated in the liver of piglets challenged with LPS. This suggested that LPS did cause an inflammatory response in the liver, which was generally consistent with previous studies (31). Interestingly, dietary supplementation of NAC alleviated the up-regulation of IL-1β caused by LPS. Additionally, the levels of IGF-1, total GSH and cysteine in the liver were elevated with NAC treatment. It has been previously mentioned that NAC might alleviate LPS-induced tissue injury by improving antioxidant function, which might also be characterized in the liver. In addition, IGF-1, a growth- promoting factor, played a key role in the growth and anabolism of tissues (32). This suggested that NAC might alleviate LPS- induced tissue injury by promoting its growth, as found in the present study, NAC raised the level of EGF and the ratio of total protein to DNA in the liver. In the present study, treatment with NAC up-regulated the mRNA levels of *HIF-1, Nrf-2*, and *IGF-1* in the liver. Moreover, the level of total cysteine was positively correlated with that of IGF-1, whereas the content of IL-1β was negatively correlated with the levels of total GSH and the ratio of total protein to DNA in the liver. These findings provided further evidence for our hypothesis that NAC might alleviate LPS-induced tissue injury by regulating multiple targets, such as, Nrf-2/HIF-1, EGF, and IGF-1 signaling pathways.

Another interesting finding was that the transcription levels of genes related to type I interferon pathway were up-regulated with LPS treatment. Additionally, the mRNA levels of *CXCR3, HSPB1*, and *IFN-*γ were also up-regulated. The type I interferon signaling pathway was involved in the antiviral response and in regulating inflammation to maintain immune homeostasis (33). In this study, dietary supplementation with NAC further promoted the transcription of genes related to the type I interferon signaling pathway, as was also observed in our previous study (12, 34). It illuminated us that NAC might improve the immune function of piglets by regulating type I interferon signaling pathway. CXCR3 induced the proliferation of type Th1 cells, which were pro-inflammatory cells, and mediated the migration of immune cells to the site of infection, thus causing lymphocyte infiltration. CXCR3 expression was up-regulated in the inflammatory response (35). IL-1β and IFN-γ were representative cytokines secreted by type Th1 cells, which were involved in regulating the body's immunity. Moderate levels of IFN-γ helped stimulate the immune system, and its expression was up-regulated in an inflammatory state (36). Study also suggested that HSPB1 was regarded as an inflammatory marker gene in a variety of diseases (37). Notably, in this study, NAC alleviated the LPS- induced up-regulation of *CXCR3, HSPB1*, and *IFN-*γ. Those outcomes demonstrated that NAC might inhibit the polarization of Th cells to Th1 cells to inhibit the expression of pro-inflammatory cytokines, and it might need to be mediated by type I interferon signaling pathway. We firmly believed that the mechanism here was worth further study in the future.

**Figure 6:**
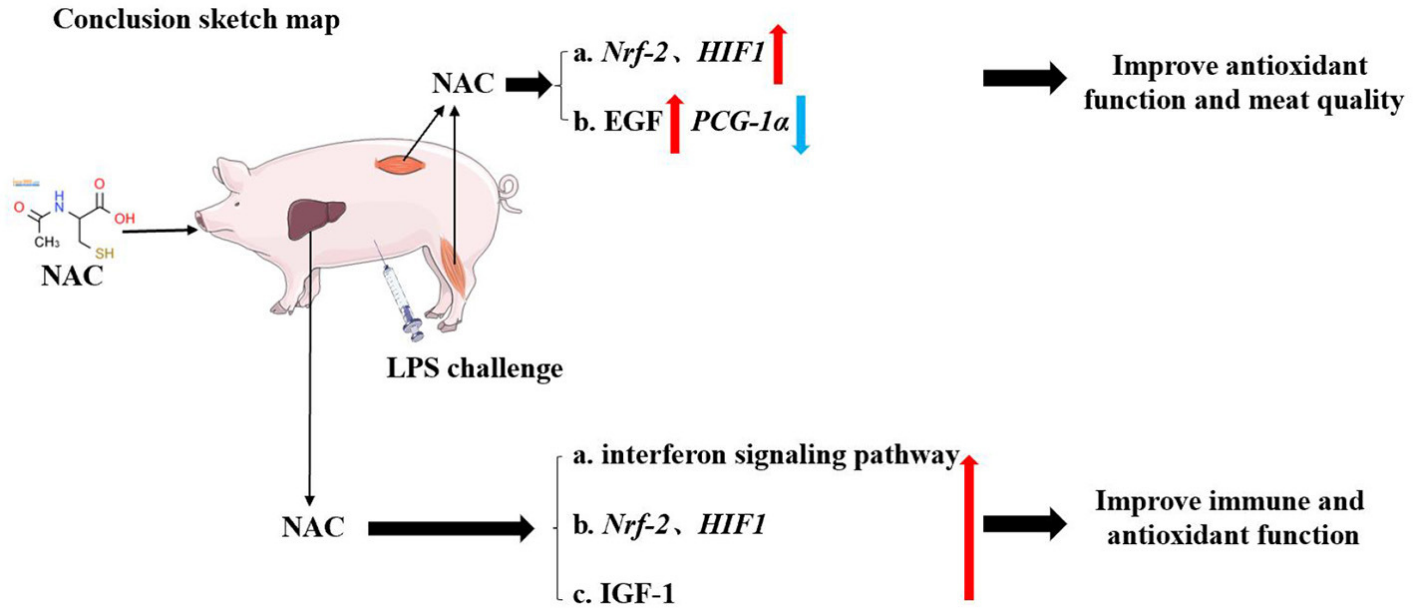
The schematic diagram of our conclusion. Dietary supplemental NAC might alleviate the negative effects of LPS on muscle by activating the Nrf-2, HIF-1, and EGF signaling pathway. It also improved the quality of muscle by regulating *PGC-1*α gene. NAC also alleviated LPS-induced liver injury in piglets by regulating HIF-1, Nrf-2, IGF-1, and type I interferon signaling pathways.

Finally, the outcomes of the correlation analysis showed that the levels of IGF-1, total GSH and cysteine, as well as the transcription levels of *Nrf-2* and *HIF-1* were positively correlated with genes related to the type I interferon signaling pathway. This suggested that the protective effects of NAC on the liver and muscles of piglets did not depend solely on a single regulatory target. In general, NAC could mitigate the adverse effects of LPS on the liver and muscle through multiple pathways, for instance, Nrf-2/HIF1, EGF, IGF-1, and type I interferon signaling pathways (Figure 6), which were also closely synergistic with each other.

## 5 Conclusion

Dietary supplementation with NAC alleviated the negative effects of LPS on muscle by improving antioxidant function and promoting tissue growth. It might also improve meat quality by regulating the *PGC-1*α gene. Additionally, NAC alleviated LPS- induced liver injury in piglets by regulating Nrf-2/HIF-1, EGF, IGF-1, and type I interferon signaling pathways.

## Data Availability

The original contributions presented in the study are included in the article/supplementary material, further inquiries can be directed to the corresponding author.
